# Supplementary material for: Qualitative and quantitative hard-tissue MRI with portable Halbach scanners
Source: Sci Rep. 2026 Jun 15;16:27343. doi: 10.1038/s41598-026-56727-8 (PMC13530120; doi:10.1038/s41598-026-56727-8)
Supplement: Supplementary file 1 [file 41598_2026_56727_MOESM1_ESM.pdf]

Supporting Information for: Qualitative and quantitative hard-tissue MRI with portable Halbach scanners

Jose Borreguero*, Luiz G. C. Santos*, Lorena Vega Cid*, Eli G. Castanon*, Marina Fernández-García*, Pablo Benlloch*,†, Rubén Bosch*, Jesús Conejero*, Pablo García-Cristóbal*, Alba González-Cebrián*, Teresa Guallart-Naval*, Eduardo Pallás*, Laia Porcar*,‡, Lucas Swistunow*, Jose Miguel Algarín*,†, Fernando Galve* and Joseba Alonso*

*MRILab, Institute for Molecular Imaging and Instrumentation (i3M), Spanish National Research Council (CSIC) and Universitat Politècnica de València (UPV), 46022 Valencia, Spain

†Full Body Insight S.L., 46980 Paterna, Spain

‡PhysioMRI Tech S.L., 46980 Paterna, Spain

TABLE SI
ACQUISITION PARAMETERS FOR PETRA SEQUENCES.

Image	FA (°)	RF pulse length (µs)	FoV (cm^3)	Pixel size (mm)	Dead time (µs) / Acquisition time (ms)	Bandwidth (kHz)	TR (ms)	US_{rad}	Radial spokes	Pointwise spokes	Averages	Scan time (min)
Fig. 3a.I	90	4/20/3	20×16×16	2×2×2	150 / 1	50	50	1	25,250	568	1	21.5
Fig. 3a.II	90	4/20/3	20×16×16	2×2×2	175 / 1	50	50	1	25,250	1,584	1	22.4
Fig. 3a.III	90	4/20/3	20×16×16	2×2×2	200 / 1	50	50	1	25,250	3,416	1	23.9
Fig. 3a.IV	90	4/20/3	20×16×16	2×2×2	200 / 2	50	50	1	25,250	488	1	21.4
Fig. 3a.V	90	4/20/3	20×16×16	2×2×2	200 / 3	50	50	1	25,250	168	1	21.2
Fig. 3b.I	90	4/20/3	20×16×16	2×2×2	200 / 1	50	50	1	25,250	3,416	1	23.9
Fig. 3b.II	90	4/20/3	20×16×16	2×2×2	200 / 1	50	50	2	12,622	3,416	1	13.4
Fig. 3b.III	90	4/20/3	20×16×16	2×2×2	200 / 1	50	50	4	6,370	3,416	1	8.2
Fig. 3b.IV	90	4/20/3	20×16×16	2×2×2	200 / 1	50	50	8	3,226	3,416	1	5.5
Fig. 3b.V	90	4/20/3	20×16×16	2×2×2	200 / 1	50	50	16	1,604	3,416	1	4.2
Fig. 3c.I	90	4/10/3	20×16×16	2×2×2	200 / 1	50	50	1	25,250	3,192	1	23.7
Fig. 3c.II	90	4/20/3	20×16×16	2×2×2	200 / 1	50	50	1	25,250	3,416	1	23.8
Fig. 3c.III	90	4/50/3	20×16×16	2×2×2	200 / 1	50	50	1	25,250	4,176	1	24.5
Fig. 3c.IV	90	4/150/3	20×16×16	2×2×2	200 / 1	50	50	1	25,250	7,512	1	27.3
Fig. 3c.V	90	4/300/3	20×16×16	2×2×2	200 / 1	50	50	1	25,250	15,096	1	33.62
Fig. 4c&d	90	4/20/3	20×16×16	2×2×2	200 / 1	40	50	1	25,250	1,936	1	22.6
Fig. 6a	10:10:90	4/20/3	20×16×16	2×2×2	300 / 1	50	10	5	5,108	10,600	2	5.2
Fig. 7 top	40	2.5/20/2.5	20×16×16	1.6×1.6×1.6	300 / 1.25	48	20	4	9,488	10,752	2	13.5
Fig. 7 bottom	30	2.5/10/2.5	20×16×12	1.6×1.6×1.6	320 / 1.25	48	25	4	7,086	9,096	2	13.4
Fig. 8a&b.IV	30	2.5/20/2.5	20×16×16	2×2×4	200 / 1	50	50	2	6,380	1,864	1	6.8
Fig. 8c&d.IV	40	2.5/20/2.5	20×16×12	2×2×3	300 / 1	50	20	4	3,232	5,151	3	8.4
Fig. 9a	20:20:80	4/20/2.5	20×16×16	2×2×4	200 / 1.0	48	25/50/100	4	3,232	1,832	4/2/1	8.4
Fig. 9b&10a	-	4/20/2.5	20×16×16	2×2×4	200 / 1.25	48	50	4	3,842	3,568	1	6.1
Fig. S2	30	0/25/0	24×24×24	3×3×3	300 / 1.5	26	100	5	4,088	2,800	1	11.4

TABLE SII
ACQUISITION PARAMETERS FOR RARE SEQUENCES.

Image	Pulse time (µs)	FoV (cm^3)	Pixel size (mm)	TI (ms)	TE (ms)	ETL	TR (ms)	T_{acq} (ms)	Dummy pulses	Partial Fourier (%)	Averages	Scan time (min)
Fig. 5a	28/56	20×16×16	2×2×4	0	20:100	4	800	4	0	70	1	7.4
Fig. 5b	32/64	20×16×16	2×2×4	0:600	20	4	800	4	0	70	1	7.4
Fig. 8a-d.I	100	20×16×16	2×2×4	0	15	4	800	2	0	70	1	7.5
Fig. 8a-d.II	100	20×16×16	2×2×4	0	40	8	800	2	0	70	2	7.5
Fig. 8a-d.III	100	20×16×16	2×2×4	0	20	4	120	2	10	70	7	7.8

TABLE SIII
SUMMARY OF RESIDUAL ARTIFACTS AND SEQUENCE OPTIMIZATION EFFECTS IN LOW-FIELD PETRA ACQUISITIONS, INCLUDING THEIR PHYSICAL ORIGIN, MANIFESTATIONS, AND MITIGATION STRATEGIES.

**Artifact Source**	**Image Manifestation**	**Mitigation Strategy**	**Residual Limitation**
Tx/Rx Switching Gap (T_{dead})	Central lobe resembling a sinc function due to residual ring-down energy.	Adjustment of T_{dead} (175–300 μs) based on signal amplitude.	Trade-off between SNR loss (long T_{dead}) and ring-down artifacts (short T_{dead}).
T_2^* Relaxation ($T_{acq} > T_2^*$)	Blurring, anisotropic resolution loss, and hyperintense banding.	Keeping T_{acq} close to system T_2^* ($\approx$ 1 ms) and precise shimming.	Fundamental T_2^* limit is intrinsic to B_0 inhomogeneity distribution.
Radial Undersampling (US_{rad})	Loss of nominal resolution and degradation of fine structural details.	Selection of moderate US_{rad} factors (up to $\times$4) for large structures.	Significant resolution loss at high acceleration factors ($\times$8, $\times$16).
Long RF Pulses (t_{RF})	Sinc-like radial brightness modulation and off-resonance blurring.	Use of short, high-bandwidth RF pulses ($<$ 20 μs).	Peak power (B1) limitations of the GPA may constrain minimum t_{RF}.
Gradient Instabilities / GPA	Waving patterns of varying orientations in the FoV.	Hardware-level monitoring and stabilization.	Minor current fluctuations still produce subtle periodic patterns.
Voxel Dimensions (Slice Thickness)	Partial volume effects masking small anatomical structures.	Optimization of T_{scan} vs. resolution trade-off.	Slice thickness limited by clinically viable scan times.
Gradient Non-linearity	Hyperintense bands at the boundaries of the imaging volume.	Model-based (CP) reconstruction.	Persistence of artifacts at the edges of the linearity volume.
Coil Hardware (External)	Circular and vertical halos (axial and coronal/sagittal planes).	Identification as non-intrinsic (PLA holder structure).	Visualization of physical setup due to coil proximity.

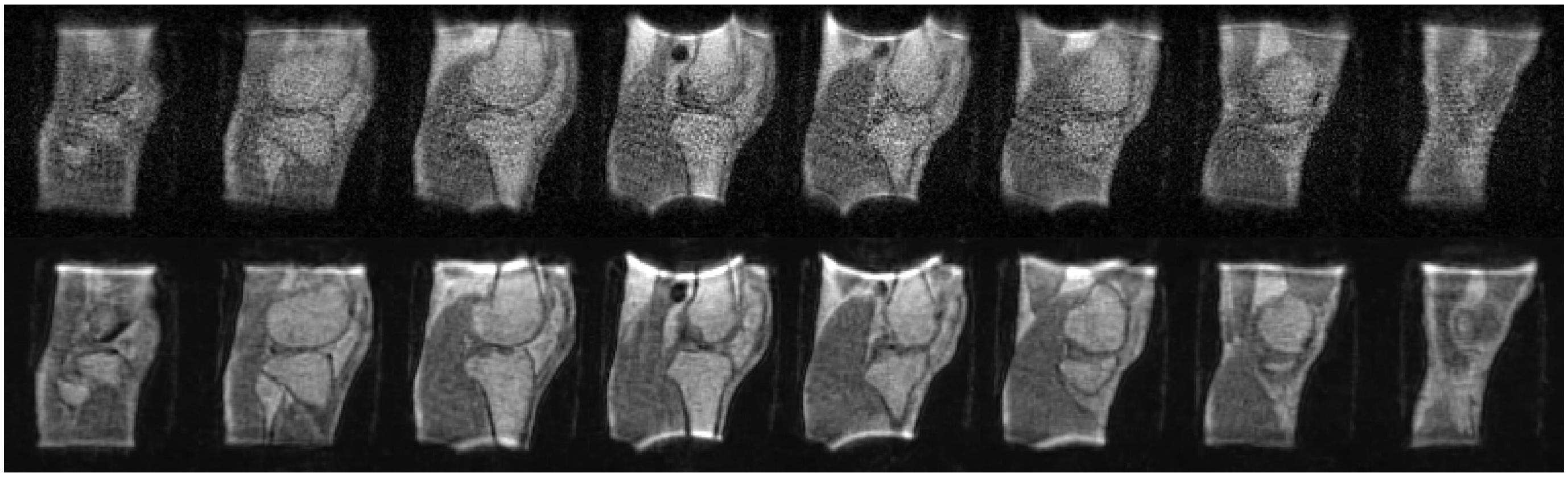

Fig. S1. Representative z-slices of the knee PETRA dataset shown in Fig. 7 but reconstructed using two different approaches: (top) a standard FFT-based reconstruction, where radial k-space spokes were interpolated onto a Cartesian grid without density compensation prior to the inverse FFT; and (bottom) Algebraic Reconstruction Technique (ART) incorporating $B_0(r)$ and $B_1(r)$ prior knowledge by means of a prior SPDS mapping. Both are shown here without retrospective denoising.

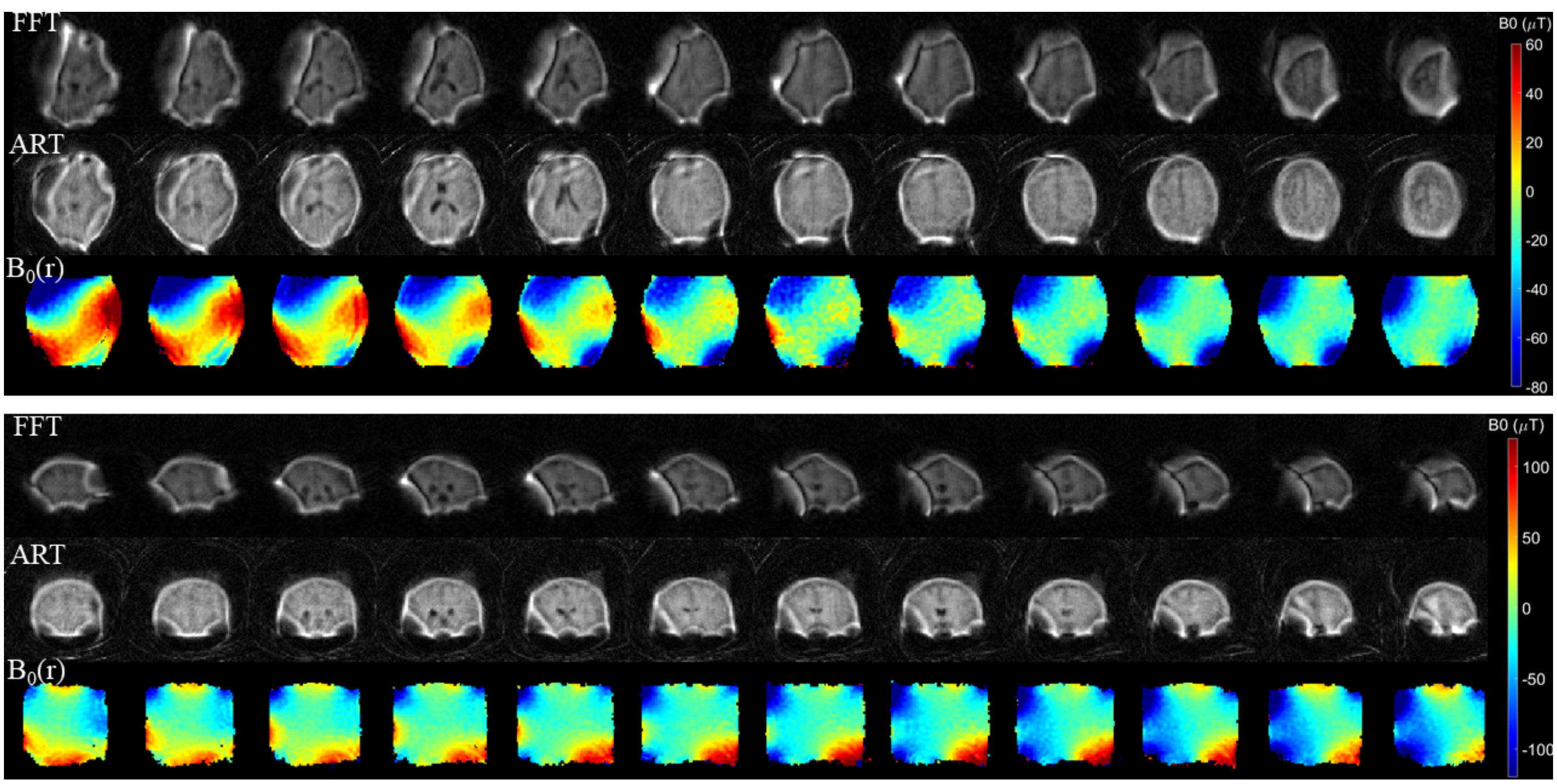

Fig. S2. Axial (top) and coronal (bottom) slices of a human brain acquired with PETRA on the 90 mT Halbach scanner. The upper rows show the FFT reconstruction, whereas the middle rows display the model-based ART reconstruction. The bottom rows present the $B_0(r)$ maps obtained using SPDS with a customized phantom (filled with a 16% $CuSO_4$ aqueous solution) designed to occupy a volume equivalent to that of the human head. The measured spatial B_0 information was subsequently fitted to a sixth-order polynomial function and incorporated into the encoding matrix (Eq. 12) used for the ART reconstruction.
